# The Baby’s Second Summer*Read before Brazos Valley Medical Association, at Caldwell, May 8, 1900.

**Published:** 1900-08

**Authors:** E. S. Ferguson

**Affiliations:** Cameron, Texas


For Texas Medical Journal.

### The Baby's Second Summer.*

BY DR. E. S. FERGUSON, CAMERON, TEXAS.

The baby, after passing through the first year of its life, may find that troubles have just begun. At this time the little fellow is taken from the mother's breast and often finds that to live it must hustle for itself. The idea of most people that a child can digest anything and everything as soon as it has outgrown an exclusively milk diet, leads very often to disastrous results. In taking up the subject of this paper, I will only touch lightly upon the actual troubles or diseases so common to this period, but will more especially deal with what I consider the best method of preserving a healthy, vigorous body to battle with the later years of life.

The proper time to wean an infant has been a subject of much discussion, but a few rules may be laid down, which, if carefully followed, will aid us in the majority of cases.

*Read before Brazos Valley Medical Association, at Caldwell, May 8, 1900.

In the healthy infant, about the time of the evolution of the eight incisor teeth and the appearance of the anterior molars, the glandular structures become more active, muscular tonicity increases and the alimentary tract is in a condition to digest other articles of food than milk. Then again, the mother's milk may disagree with the infant, making it necessary to wean early, but in such a case milk in some form should be the only diet given. If the hot summer months come about the time for weaning, it will be better to wean early or to wait until the summer is passed, nothing else interfering. Disease of the mother may necessitate taking the child from the breast. Very often when the mother becomes pregnant, weaning becomes a necessity, because either the child suffers from insufficient nourishment or the life of the fœtus is endangered from the same cause, or the constant irritation of the breast may bring on contractions of the pregnant uterus. Let us urge all mothers, unless there is some good reason otherwise, to nurse her child until it reaches the age of from twelve to fourteen months. If it becomes necessary to wean earlier, or if hot weather is at hand, even though the functions of digestion be well developed, the diet should be restricted to milk, with the possible exception of a little barley water or egg albumen. In most cases a strictly milk diet is to be preferred. In taking the child from the breast a gradual change will in most cases give the least trouble. From day to day lengthen the nursing period, substituting a suitable food (milk by all means if possible), until you have found that the child thrives, when it can entirely be taken away with safety.

In starting the child out in life, outline a regular bill of fare for each meal, always remembering that simplicity must be the watchword; fancy dishes, candies, sweets, etc., should most certainly be discarded. Watch carefully your change of diet and by close observation add or drop articles from your list, as indications of trouble present themselves. Regular hours for feeding is necessary during the second year, more particularly during the hot summer months. I would advise five meals a day, making the regular three meals a more or less mixed diet with milk the other two, one between breakfast and dinner, the other between dinner and supper.

The problem of carrying the child through the second summer, when you are making probably, almost a complete change in its mode of living, when the hot weather predisposes to stomach and bowel troubles by favoring fermentation and bacterial diseases, is by no means an easy one.

Let us, as physicians, urge upon our patrons the necessity of careful feeding, careful nursing, careful handling, dressing, bathing and exercise. After selecting the most favorable diet in which all the constituents necessary to growth and development, proteids, fats, carbohydrates, mineral salts and water, are present, use every effort to administer it in the most intelligent manner. Very often we have to deal with a stomach which has been abused from early infancy by careless feeding, over-distension, etc. If such is the case, ours is of necessity a harder lot to fill, and more rigid must be the rules laid down for its daily care.

How often have we all been called in, to find a babe during its second summer with a pinched expression, sunken eyes, moaning and fretful, fever, vomiting everything placed in its stomach, bowels either running off or severely constipated, losing flesh daily, the mother wringing her hands, telling you that she cannot understand why her darling baby is so sick. On inquiry you will find that she has probably weaned her baby four or six weeks previously and has made no provision whatever for its diet, but takes her child to the table with her and allows it to eat or drink any and everything it wishes or sees, and between meals to go around with a biscuit in one hand and a hunk of meat or piece of cake in the other; no wonder half of our cases of indigestion, gnawing the very life out of our adult patients, can trace their troubles back to indiscretions in early infancy. Until the child has reached at least its fourth year the parent should select everything which passes the child's lips in the shape of food or drink, and if it has been guarded carefully and intelligently, in the great majority of cases, I will show you a healthy child, and in later years a happy man or woman. Constipation is a very frequent ailment in the second summer of a baby's life, caused most frequently by either unsuitable food, abuse of medicine, or digestive derangements. During the nursing period nature furnishes the child in almost all cases a fixed ratio in the elements of nutrition, and until the child grows older and its daily life becomes more active, we aim to prserve this ratio in the food element, or the bowels will rebel and derangement inevitably result. Fats in the proper amount (about four per cent.) act as a natural lubricant to the alimentary tract and an increase or decrease will increase or diminish the bowel activity, as the case may be. Other articles of food act through their irritating properties and in no way aid in nutrition. Abuse of medicine is a frequent cause of this aggravating trouble and refers chiefly to a too frequent and liberal use of laxatives or to the use of opiates and other soothing remedies. The former of these medicines produces the constipated habit by changing the natural secretions of the bowels and exhausting their usual expulsive action; the latter by drying up the secretions and paralyzing the peristaltic action of the bowel. Advise the mother that it is much safer and better to use some simple mechanical means to overcome the trouble than to disturb the nutrition and predispose the infant to rectal troubles of various kinds. Digestive derangement brought about by an alteration in the digestive secretions due to disease, such as malaria, nausea, sore mouth, liver troubles, etc., will often produce constipation. In treating this affection find out the cause, stop the troublesome medicines, arrange the diet to suit your patient, as nearly as possible, and use some simple means to relieve the bowels temporarily, and then guard against a repetition. Do not disturb the whole alimentary tract, if possible stimulate the lower bowel by small enemas of soapsuds or by using a glycerine suppository. If this fails, use a little olive oil or small medicated injection of Epsom salts and water, which will act fully as well as if swallowed. In these cases, if it is necessary to give a laxative, use milk of magnesia with the food, and you will generally be rewarded by happy results.

Very often we find a child suffering with griping and diarrhœa during this important period in life, generally caused by improper food, fermentation or irritants. In these cases study the cause, arrange the diet and avoid that common practice among physicians of giving the routine dose of calomel to each and every patient they are called to see with this trouble. Often after careful supervision of the food and probably flushing of the lower bowel with a hot saline solution, the trouble will disappear. This may be aided by administering a mild digestant, such as pepsin and sulphocarbolate of zinc, with or without some of the mineral acids. The nervous system also should receive special attention from the family physician, for it is a lamentable fact that there is a steadily increasing number of young children suffering from functional nervous diseases. During the first two years the brain grows more than in all the rest of life; hence the necessity of insisting on rest, sleep and absence of all excitement. How often do we find parents and relatives continually playing with and exciting the young child in various ways without even a thought of injury. Let us remember that the instability of the nervous centers and the extreme irritability of the motor, sensory and vaso-motor nerves, caused by the rapid growth of the brain and cord cannot stand the strain of excitement and hilarity. Mothers and fathers will tell you that they cannot see how that will hurt the child; no, they cannot, but, gentlemen, it surely does do so. Why do adhesions about the prepuce, worms, slight disturbances, indigestion, eye-strain, adenoid growths, fever, etc., bring on very often extreme nervous conditions? Simply because the weak nervous system is unable to stand the strain. Then why should we be so careless in stimulating to excitement in other ways? Do not permit it! Also avoid the use of all stimulants, tea, coffee, alcohol, etc.; they do not help nutrition in any way, and do much harm to the nervous system. Insist on plenty of sleep, at least thirteen or fourteen hours for a child between one and two years of age. Bathing with tepid or cool water each morning should be made a regular practice.

In closing, I would recommend that physicians spend more time in instructing the mothers in the proper care of their children, and less on dosing the little fellows with medicine.

